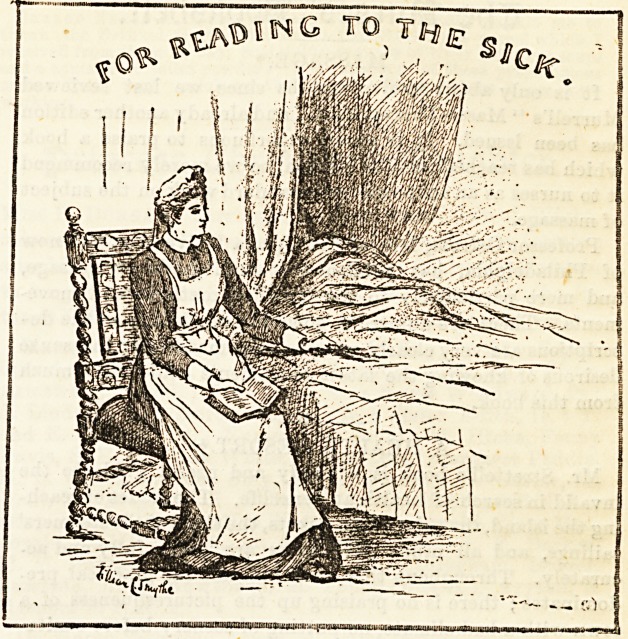# The Hospital Nursing Supplement

**Published:** 1891-04-18

**Authors:** 


### "The Hospital" Nursing Mirror.

Being the Extra Nursing Supplement of "The Hospital" Newspaper.

Contributions for this Supplement should be addressed to the Editor, The Hospital, 140, Strand, London, W.C., and should have the word "Nursing" plainly written in left-hand top corner of the envelope.

### En Passant.

ROMAN CATHOLIC NURSES.—The Ladies' Committee at Basingstoke have dismissed the District Nurse on discovering that she was a Roman Catholic; the advertisement was for a "Christian woman," and the action of the Committee has been severely criticised. The Matron of the Norfolk and Norwich Hospital has lately joined the Roman Catholic Church, much to the consternation of the Committee of Management, but they have stopped short of calling upon her to resign.

KENT INSTITUTION.—The annual meeting of the Kent Nursing Institution was held at the Lady Caroline Neville Home at West Malling. The chair was taken by the Hon. Mary Boscawen, and there were also present the Viscountess Torrington, Lady Isabel Bligh, and Lady Logan. The Hon. Secretary, the Rev. W. Gardner Waterman, read the report. The committee state that the good progress both in work and usefulness of the Institution still continues. The earnings of the nurses are £530 in excess of their earnings in 1889. There have been 1,558 weeks of nursing, of which 133 weeks have been gratuitous or on reduced terms. The number of nurses on the staff is 45, and their skill is worthy of all commendation.

SHORT ITEMS.—Mrs. Royle and Miss Williams, nurse and assistant nurse at Fulwood Union, have been called upon to resign owing to having neglected to supply convalescent patients with clothing, and thus keeping them in bed for weeks after the doctor had ordered them up.—The *Scottish Leader* is producing a series of articles on "Sick Nursing in Edinburgh."—Nurse Coxall, of Penzance Nursing Association, attended 99 cases last year amongst the poor of the town.—The College of Physicians has reported in favour of legislation for midwives, and has urged the formation of a Select Committee.—Sister Ruth, of Hampton Wick, has visited 95 cases during the last year, 23 of which were midwifery cases. Sister Ruth's services are greatly appreciated.—Miss Kate Marsden has arrived at Tobolsk.—The new dining hall for nurses at the Edinburgh City Hospital was lately opened with ceremony in the presence of a large gathering of ladies and gentlemen.—Miss Florence Nightingale has consented to join the Council of the International Congress of Hygiene.

THE LATE MISS FISHER.—For the third Easter in succession the nurses of the Philadelphia Hospital visited Woodlands Cemetery and laid flowers on the grave of Miss Alice Fisher, the English nurse, who died in June, 1888. Within the last two weeks a granite slab, the gift of Mr. G. W. Childs, has been laid on the grave. The slab is quite plain, with the exception of a Winchester cross in relief at its head, and this inscription: "Alice Fisher, born Queen's House, Greenwich, England, June 14th, 1839; died Philadelphia Hospital, June 3rd, 1888." The nurses, thirty in number, set out for the cemetery in procession, headed by the hospital chaplain. On reaching the grave portions of the Burial Service were read, and the hymns, "The King of Love my Shepherd is," "The Strife is O'er, the Battle Done," and "For All the Saints," were sung. After the benediction the nurses strewed lilies, azaleas, and other Easter flowers on the grave, which was almost hidden by the white blossoms. The site of Miss Fisher's grave is a lot purchased by her friends. Mr. Childs yesterday announced his intention of buying an adjoining lot for the burial of nurses who may die in the service of the Philadelphia Hospital.

BRISTOL NURSES' SOCIETY.—Mr. John Harvey presided at the annual meeting of this society on April 6th. There was a good attendance. The annual report spoke of three new districts undertaken in the year; Miss Corneille and Miss Theresa Lucas came in for special praise for their earnest and loyal work. The financial statement showed a deficit of £244. The High Sheriff (Mr. J. H. Lockley) remarked that if the society had been enabled to do the amount of good represented by an attendance on 2,000 families with a number of visits exceeding 60,000, much more could have been accomplished with larger funds at its disposal. Bristol was celebrated for its philanthropy, and he was sure that directly it was known that that excellent society was in need it would at once be supplied, not only with the amount necessary to wipe off the deficit, but to enable them to increase their nursing operations generally.

NORTHERN NURSING ASSOCIATION.—A public meeting was held at Manchester, in the Town Hall, last week, in support of the Northern Branch of the Workhouse Infirmary Nursing Association. The objects of the Association are (1) to promote the employment of trained nurses in workhouses, (2) to supply trained nurses to Boards of Guardians requiring them, and (3) to train nurses specially for the work. Miss Wilson, Hon. Secretary of the Southern Association, gave an account of its work. They had tried, she said, to cover the whole of England, but it was too large a task, and they were very glad that the Northern Association had come to their relief. The Chairman, the Right Hon. J. T. Hibbert, spoke in favour of the movement, and a very influential committee was appointed for the year. The meeting was in every way most satisfactory, and amongst others present were Lady Edward Cavendish and Sir H. E. Roscoe, M.P.

NEW INVENTIONS.—We have received from Messrs. Fannin and Co., of Dublin, a clinical slate arranged for the use of nurses in private houses. The slate is carefully ruled in spaces for the noting of the temperature, pulse, diet, &c., of the patient during twenty-four hours, and can then be cleaned and used again. We have also received from Messrs. Gilbertson and Sons, of Holborn Circus, a chart for the recording of the doctor's instructions, designed by Nurse Marian Pincoffs. This chart is very full, going into the question of the position of the patient, whether to be roused for medicine, in what cases the doctor is to be summoned, and so on. In very serious cases, where the nurse is not experienced, it would prove very useful. It is Messrs. Gilbertson and Sons, who prepare the Ung. Emolliens Antisepticum which nurses find so useful in preventing and healing cracks in the hands.

AN EXTRAORDINARY REPORT.—Our attention has been called to an inaccuracy contained in the last annual report of the Trained Nurses' Annuity Fund. It is there stated that the "so-called National Pension Fund for Nurses is only an insurance company, and does not meet the cases of poor broken down nurses." How such a statement could have appeared in connection with a fund which was desirous of being absorbed by the Royal National Pension Fund some years ago we do not understand. Its ludicrous inaccuracy will become apparent when we point out that the income arising from the Trained Nurses' Annuity Fund is about £200 per annum, whereas the income derived from the Benevolent Fund of the Royal National Pension Fund alone is £450 per annum. Again, the total invested property of the Trained Nurses' Annuity Fund amounts to less than £5,000, whereas the Pension Fund has a Benevolent Fund of £10,000, and a Donation Bonus Fund of £40,000 in addition. We are quite sure that this statement was made without the knowledge or the consent of H.R.H. the Princess Christian, and why the writer, whoever he or she may be, should have gone out of the way to make so false a statement we are at a loss to imagine.

### Male Nurses.

(By One of Them).

(*Concluded from page* x.)

In male nursing one often hears objections raised to men being employed in the house in this capacity. In many places they are looked upon as a dangerous and foreign element, and it is generally thought that they are not willing to make themselves useful beyond what they engage for. I doubt very much whether this can be maintained when good male nurses are engaged; we often hear of great and glaring defects, but if looked into, this is chiefly on account of the loose way men are employed as male nurses. A case bearing on the above that came under my notice may be of interest, whilst it also tends to prove my assertion. A difficult case of spinal meningitis with complications required about fourteen months' male nursing from first to last, was taken for the last six weeks of that time by a good male nurse; it was a case requiring skill, care, tact, and unremitting attention. There had been six different men previously employed, neither of whom were male nurses, notwithstanding that from the first the family doctor had recommended that a trained male nurse should be engaged; the friends of the patient were prejudiced against men being employed in the house, but especially so against male nurses, and thought by advertising in the local newspapers, they could get men at a cheaper rate "who would be just as valuable for the nursing required, and ever so much more useful in the house when not nursing." These people were not wise, for any male nurse would be able to tell them the class of men they would be likely to get under those circumstances; but there are many like them, and fortunately for male nurses these found out they had made a mistake, and what is more, they acknowledged having done so. All these six men were not bad men, far from it, but they were all unsatisfactory as nurses; neither is that to be wondered at, for the case was extremely trying—one was a desperately bad man, and the others were unreliable in either not being able to be safely trusted with intoxicating liquors, or in not being able to awake by night, so that as a last resource a trained male nurse was applied for and obtained. Very stringent rules and agreements were made, regulations were to be observed, and the male nurse was looked upon with general suspicion in that house, but before a week was past it became evident to all concerned that all this was unnecessary; the sick-room was changed as if by transformation, the servant coming there for domestic work was gently told "She would not be required," the very irritable patient was soothed; the male nurse always appeared as if he had his work anticipated, and without any apparent effort, all was regulation and order; the doctor came with smiles and good words, and, what was best of all, the mistress of that house—and she was prejudiced against male nurses—came to acknowledge that she had been in error, and with genuine tears and fervent voice, thanked God, in the male nurse's presence, that He had seen fit to send such comfort to her afflicted husband in his last days on earth, and she often told that nurse "that money could not pay him for his devoted service, and that she was nearly ashamed to offer it him."

Respecting male nurses' general inclination for usefulness, and their requiring privileges unduly, I have never had much means of judging, further than individual experience, and perhaps it would not be right to introduce a personal element in judging a class, especially as the writer has, for much the greater part of his life, been otherwise employed than at nursing; but thus far will he go, he is always willing and glad to make himself as useful as possible, at as many different kinds of work as possible, if for no other reason, he finds his health benefits by doing so; he never, if he can avoid it, makes any hard and fast rule on taking a case, rather trying to fit himself into the different circumstances as they differently occur, trusting, and trustful that the doctor will not see him overlooked or unnecessarily overworked, and, although not a teetotaller on principle, he never takes intoxicants whilst on any duty or duties. On the other side of the question, one has to assert themselves at times. Here is an illustration: It was thought necessary that a patient and his attendant male nurse should be lodged in the country. Terms were sought, and settled, at a farm, the terms being inclusive for both. The attendant nurse's orders were strictly not to leave his patient night or day, but, unfortunately, there were no arrangements made that a local doctor should see the afflicted one, but a general order given, "that if fresh developments showed for the worse, a doctor was to be called in at once." That, from my experience, is unsatisfactory. An attendant nurse should always be under a doctor direct. Those farming people proved selfish, and wanted to make the nurse a farm labourer, and, in their idea, they do his work, and because he did not choose to enter into these arrangements, complaints were made to patient's friends "that he demurred if asked to do anything." When spoken to on the subject, he frankly and truthfully told them "that it was not true that he demurred, but that he simply did not do compulsory work on the farm, but when he could do any voluntary work, and look to the interest of his patient at the same time, he did so." He was commended for his frankness, and thanked for having so well carried out his orders, and the local doctor from that time was to see his patient at intervals. Is it not quite likely that those farming people will not speak very highly of that nurse?

Then, at the best, we know in the nature of things, one does not like men in a house, and we need not for a moment discuss their replacing, or even clashing with women as nurses; but if good male nurses—and they are limited—are selected, and paid for, I have every reason to believe they will give every satisfaction in the capacity of nurse and otherwise, well consistent with their duty; and if a period for training becomes general, passing through the ordeal of discipline, and under trained observation, will be yet another guarantee of their suitability for the work. Men at the present time very successfully nurse our soldiers and sailors in sickness. I remember in my younger days visiting the wards of the Royal Naval Hospital, at Plymouth, in the West of England; the thing that most struck both me and a friend at the time of doing so was a middle-aged man in each of the wards, dressed something after the style of a prison warder, which, upon enquiry, we found was the nurse in charge of the ward. They were not trained, other than the experience they would naturally gain from their employment, but were simply men pensioned from the Royal Navy and Marines, after serving in different capacities for their full period of 20 years. One would almost fancy it was impossible for these men to be successful or satisfactory nurses; but I suppose they were, or otherwise they would not be kept. Both my friend and I thought it strange that they did not train a body of men for nursing in the ships when at sea or in foreign countries, or in case war was to break out, when one would think they would be very valuable in the ships. I think there is some alteration in the system now, for if I remember rightly, about twelve months ago there was some reference made in the "Nursing Mirror" of a "pensioner" being likely to get into trouble for having written a diatribe against naval nursing sisters, and possibly the fact of sisters being there instead of the "pensioner" explains the whole matter. There were no nursing sisters in the hospital at Plymouth when I visited there; if there are now I should think it one of the greatest blessings that it could be possible to bestow on patients entering therein, the knowledge of being nursed under the guidance and directions of intelligent, thoroughly-trained, and in many respects self-sacrificing, nursing sisters. I hold it is a blessing to be—when the case makes it necessary—nursed by a well-trained male nurse; but, at the best, that blessing is small in comparison with the other.

### The Eastern Hospital Scandal.

This inquiry was continued last week, when Miss Aston was cross-examined. She stated that she had come to England for the express purpose of giving evidence at this inquiry. She was asked to come over by the solicitors engaged for the complainants. Dr. Bridges here stated that the Local Government Board thought it exceedingly desirable that Miss Aston should come over, and instructed him to telegraph to her. As to the food, she had never tasted a good potato all the time she was in the hospital. She complained to Dr. Collie, and he said they were so bad he could not eat them. She made complaints to Dr. Collie as to the food and clothes supplied to the nurses. She had suspicions regarding the sobriety of the Night Superintendent, Nurse Dowsett. She remembered some bother about an assistant nurse in Nurse Farnham's ward "Inventing the temperatures." She also remembered a conversation with Dr. Collie as to re-naming the wards. Dr. Collie said that if the Committee objected to the names of saints they should name the wards after themselves—"John, Maud, and Isabel." He said he would only have those three, as the rest were "swine." I resigned immediately after the dancing. The managers gave me a testimonial which was hardly complimentary. They said I had not been very successful as Matron.—Do you mean to say that Dr. Collie knowingly misled the Committee?—I think he must have done, because he must have known about the bad food, and that Nurse Dowsett was not a suitable person to be Night Superintendent. I heard about a bribe being asked for or promised to Nurse Dowsett of a pair of gloves by one of the nurses. I was told that the whole affair was a joke but I did not believe it.

Emily Niblet, the next witness, said she had been a nurse four years in the hospital. In 1890 she sent in her resignation as she had not been promoted as Charge Nurse. Dr. Collie said she was not fitted to be Charge Nurse. The resignation was accepted, but when she went to Dr. Collie for her testimonial he suggested that she should not leave. She withdrew her resignation, and was afterwards appointed Charge Nurse. Mr. Eldridge: I put it to you. Were you not going about the ward saying that it would not do to let you go? No, I was not.—Did you say that you knew a little too much about Nurse Dowsett? No, but Nurse Halkin said to me that I knew too much to go.—And after that you were promoted?—I was promoted in May of last year. Witness further stated that she went to Nurse Andrews' funeral. Superintendent Dowsett was with them. In returning they stopped at an hotel where she and the rest had something to drink. It was not lemonade. (Laughter.)

Miss Eliza Dowsett said that she had been for the past sixteen and a-half years in the employment of the managers of the Eastern Hospital, and was appointed in January, 1875, as ward servant. She served in various capacities, until ultimately she was appointed Night Superintendent. The latter post she had held for the last five years. Cross-examination: No allowance of wine or spirits formed part of her rations, but when she was ill she was ordered Burgundy. She had never organised any raffles in the hospital, as alleged, but she had collected subscriptions on behalf of several charitable movements.

James Fergusson, gate porter at the hospital, said he had held his situation for ten years, his hours of duty being from 6 a.m to 6 p.m. When Nurse Halkin left the hospital nothing took place inside the gate. About fourteen nurses were outside. They were in plain clothes. What took place outside?—Oh, that is beyond my jurisdiction. (Laughter.) Did anything occur?—I did not see anything, but I heard something like a slight hiss. Did Dr. Collie or the Matron take part in this hissing, or whatever it was?—No; certainly not. Cross-examined by Mr. Eldridge: He was instructed to allow the night nurses to pass out till one o'clock. Nurse Halkin left a few minutes before one. The nurses were allowed out by special order of the Matron, and he did not book them in or out, with the exception of one or two who had been out earlier. He had verbal orders delivered to him by an assistant nurse, who at the same time took away the "passes" for the Matron to alter. That was an unusual circumstance, and the only instance of the kind he had known since he had been connected with the hospital.

### HAPPINESS.

We all wish for happiness, but cannot agree which is the best way to secure it. We may be sure, however, that to have a sound mind in a sound body is half the battle, and we shall be wise in trying to keep them sound. It is perhaps rather difficult and unpleasant at first to be always fighting against nature, which prompts us to take the pleasure of the moment without regard to consequences. We don't care always to hold back the angry word or blow, to resist envious wishes and evil thoughts, to be careful not to eat and drink too much, or over indulge in sloth, or exercise, or pleasure. But all these "pleasant sins," however, make whips to scourge us with by and bye. The man who adds drunkenness to thirst, and the woman who out of vanity wears insufficient clothing, sow the seeds of decay and death. Scripture and common sense alike teach us to keep under our body and have it in subjection, and alike forbid the abuse of our members. We will try to take this lesson home to ourselves and learn it perfectly. God tells us it is not by ease and pleasure that we can conquer earth, but by labour and the sweat of the brow, and we know from experience that any good and glory in this life comes by pain, "And what we win and hold is through some strife." Shall we then only think of ourselves and our ease and comfort? If "Christ pleased not Himself," how ought we to expect to find life easy? We are not fashioned for perfect peace and rest in this world, however it may be in the next. If we do not bring ourselves to see this, God will send us sickness and suffering to drive it home to our hearts, and we must take it patiently as coming from Him, who does all things well, and looking beyond this world "take the Cross for glory and for guide."

### Death in Our Ranks.

We announce with regret the death of Staff Nurse Margaret McKilligan, of Aberdeen Royal Infirmary, which took place on the 3rd inst., after a very short illness. A short service was held by the Chaplain on the 6th, at which many attended. The coffin was quite covered with flowers, given by the Hon. Superintendent, sisters, students, and others.

Annie Eleanor Murcott, aged thirty years, lately a nurse of the City of London Union, committed suicide last week. The jury returned a verdict of temporary insanity.

### The Nurses' Bookshelf.

### MASSAGE.*

It is only about twelve months since we last reviewed Murrell's "Masso-Therapeutics," and already another edition has been issued. It is surely superfluous to praise a book which has reached its fifth edition, so we merely recommend it to nurses as an acknowledged standard work on the subject of massage.

Professor Ostrom, late of the Upsala University, and now of Philadelphia, has published a small guide to massage, and more particularly to the Swedish methods and movements. There are numerous good illustrations, and the descriptions are very clearly written; the masseur or masseuse desirous of knowing the latest movements could learn much from this book.

### A HEALTH RESORT.†

Mr. Strettell's book is a handy and useful guide to the invalid in search of health at Teneriffe. The modes of reaching the island, the price of the tickets, the times of the steamers' sailings, and all other particulars are given fully and accurately. Throughout this small volume the practical predominates; there is no praising up the picturesqueness of a town with a bad climate, no putting of scenery before sanitation. Nor does Mr. Strettell fall into the common error of indiscriminate praise and lavish adulation; he maintains a judicial turn of thought throughout, and warns the reader where to expect a tiring drive, and when to expect a certain amount of rain. The book is well worth perusal by doctors and nurses in the interests of their patients.

### A MEDICAL LEXICON.‡

This is a small volume, neatly bound, and compact in form. It comprises an immense amount of information, but we are doubtful if brevity is not for once too prominent a virtue. You look up "Gerontoxon," and you find it described "Arcus senilis." The ignorant person finds the second term no more comprehensible than the first, and has to turn to the letter "A" to explain the explanation. Then a "Spore" is a "Cryptogamic analogue of seed." This is distinctly going from bad to worse. A good point in the book is that help is given in the pronunciation of the terms; also the type and printing are excellent.

### NURSING IN GERMANY.§

Germany is the cradle of trained nursing, and amongst the many handbooks of the art we have been privileged to review none has pleased us better than this book by a German doctor. It is written in the simplest language, deals with the minutest details of nursing, and is illustrated by over 400 quaint but useful woodcuts. Here you see two wooden-looking deaconesses, in the ugly sun-bonnet cap which the old sisters at Charing Cross wore, changing a drawsheet. The bed is all out of perspective to permit you to see its entire surface; but though an artist would laugh at the sketch, no nurse could see it without at once comprehending what the writer desired to teach. The German deaconess seems to have many and various duties. First, her instruction deals with splints and dressings, and the means of carrying and lifting patients. Bandaging is fully described, and has some elaborate illustrations, but massage is dismissed in two pages. The chapter on operations gives a list and description of the chief surgical instruments, and instruction in the methods of administering anæsthetics and the use of the spray. Further on we have a chapter on baths and douches, and the use of water generally as a remedial agent. The transport of the wounded, care of fever cases, rules of the deaconesses' home, and other interesting subjects, are fully dealt with, and the book concludes with an excellent index.

* "Masso-Therapeutics; or, Massage as a Mode of Treatment." By William Murrell, M.D., F.R.C.P. (London: Mr. H. K. Lewis).—"Massage and the Swedish Movements." By Ostrom. Price 2s. 6d. (London: Mr. H. K. Lewis.)

† "Teneriffe: Personal Experiences of the Island." By George W. Strettell. (London: Mr. Fisher Unwin.)

‡ "Pocket Medical Lexicon," by John M. Keating and Henry Hamilton. (London: Mr. H. K. Lewis.)

§ "Krakenpflege," by Dr. Paul Rupprect. (Leipsic: Vogel.)

### THE ROYAL NATIONAL PENSION FUND.*

Dr. Potter has here told the story of the founding, rise, and growth of the Pension Fund, and he has told it in no dry, didactic way, but so as to read as an interesting story of what can be done by purpose and perseverance. The book is prettily bound, and contains portraits of the Princess of Wales (to whom it is dedicated by permission), Mr. Burdett, Lord Rothschild, Mr. Hucks Gibbs, Mr. E. A. Hambro, Mr. Walter H. Burns, and the late Mr. Junius S. Morgan. There are also some picturesque drawings of the Marlborough House reception by Miss Lilian C. Smythe. Perhaps there is no truer sentence in the book than that in which Dr. Potter says: "If the nurses were paid according to the real worth of their services, the earnings of the best of them would rank with those of prima donnas, and not, as they do now, with those of second-rate cooks."

* "Ministering Women." By George W. Potter, M.D. (The Hospital, Limited, London.) Price 1s.

### Everybody's Opinion.

*[Correspondence on all subjects is invited, but we cannot in any way be responsible for the opinions expressed by our correspondents. No communications can be entertained if the name and address of the correspondent is not given, or unless one side of the paper only be written on.]*

### THE SECOND THOUSAND.

"One of the Second Thousand" writes: I am delighted to see in The Hospital that H.R.H. the Princess of Wales intends to receive us when the number of two thousand members is reached. Although some of us may have been envious of the privileges already conferred on the First Thousand, yet we cannot but esteem the Princess's intention a most generous one in thus extending her kindness even to the Second Thousand. We shall anticipate the coming event with great pleasure. Those hospitals which are meditating affiliation had better hurry if they wish their nurses to be in the Second Thousand.

### THE EIGHT HOURS BILL.

"Mechlenburgh" writes: Here is a comparative table, in which it is shown that the "three-shift" system (by which each nurse would have only eight hours on duty in each twenty-four) can be so worked as to cost actually less, while employing a much larger staff.

Suppose a hospital on the old "two-shift" system with ten wards, and one day-nurse, one probationer, and one night-nurse to each ward, the expenditure might be roughly as follows:—

| | Salaries. | | | |
|---|---|---|---|---|
| 10 Staff Nurses (at £25) | £250 | Food, | each | £17 |
| | | Laundry | ,, | 4 |
| | | Uniform | ,, | 4 |
| | | | £25 × 10 | = £250 |
| 10 Probationers (at £10) | £100 | ,, | ,, | 250 |
| 10 Night Nurses (at £25) | 250 | ,, | ,, | 250 |
| 3 Supernumerary (at £25) | 75 | ,, | ,, | 75 |
| 2 Receiving (at £25) | 5 | ,, | ,, | 50 |
| | £725 | + | | £875 |

For a staff of 35, total £1,600

On the "three-shift" plan a hospital would require for ten wards, two day nurses, two probationers, and one night nurse for each ward as follows (but the salaries must be a little lower, and the probationers must pay 10s. a-week for the first twelve months, but receiving food, laundry, and uniform for the whole of the first year) :—

| | Salaries. | Food, &c. |
|---|---|---|
| 20 Staff Nurses (at £20) | £400 | £500 |
| 20 Probationers (nil) | — | 500 |
| 10 Night Nurses (at £20) | 200 | 250 |
| 3 Supernumerary (at £20) | 60 | 75 |
| 2 Receiving Nurses (at £20) | 40 | 50 |
| | £700 + | £1,375=£2,075 |
| Deduct payment by 20 probationers 10s. a week each for 12 months | | £26 × £20=£520 |
| For a staff of 55, | | total £1,555 |

CRUELTY.

"E. DE C." writes: Will you permit me a small space in your valued paper to plead in behalf of the private nurse spoken against in your issue of the week before last. I know some are certainly unworthy the title of nurse, but they are few, and I always think when we hear the friends of a patient speak in strong terms of the cruelty, &c., of a nurse that we must receive the statements "cum grano salis," as many people are prejudiced against the nurse before she enters the house, and when the poor thing arrives she can do nothing right. If these people see a little steam from a poultice they immediately declare the patient is scalded, although he or she may affirm to the contrary. If a nurse refuses to remain with a patient day and night for a week when a case is not urgent she is declared to be "selfish." A friend of mine once went to a case. When she asked where she would sleep, she was told there was no bed for her, but these kind, thoughtful people said, "We have arranged to do the day work ourselves, and you must take the night; then you can go to bed in the housemaid's bed when she gets up." She was even expected to use the same sheets. Another comrade of mine was kept on duty four days and four nights without rest in a trivial case. There was no bed provided for her. I know of another nurse who went to a case for night duty. The first night there was no refreshment for her, so the second she meekly asked for a little tea and a piece of bread and butter. The mother of her patient stared at her, saying, "Have you not had your supper? It is ridiculous, and I don't know why you want to eat and drink during the night when you are well. I certainly did not expect a hospital nurse to come here and give trouble." A lady the other day told me that a friend of hers said to her, before a nurse came to her house, "My dear, remember a nurse only *requires* three, or at most four, hours' rest in the twenty-four, so don't be imposed on. They are trained to take only a little sleep." It seems some people look on us as hardly human, like a rough fellow in an infirmary who said to a nurse, "You're not a woman, you're only a nurse." In these days I own that in some ways there is far too much fuss made about nurses, and the sooner this craze is over the better for all those who have entered the profession in a right spirit.

A NURSE'S HOURS.

"A. C." writes: How sad it is nowadays to hear so much discontent amongst nurses. There seemed hopes of better things a year or two ago, but now, much to the disgrace of our profession, one scarcely picks up any paper without reading some hospital scandal. How much better if these so-called ladies would seek a more refined occupation (if they can), and leave our noble work alone. My long experience of matrons is a very happy one, and my opinion is they are, as a rule, very good women. I myself was trained in a provincial hospital. The work was hard; but at the back there was a matron, strict but full of motherly kindness for those around her, and I can conscientiously say her nurses were only too glad to assist in a little extra work, which was never her fault. Let each one do their best, and work with higher and purer motives, ever mindful of an all-seeing Eye resting on them. There are difficulties in every option of life, but make the best of them, and, though now I am just made a Matron, I glance back with joy at my training days, and sorrow that they are nearly over.

"A. E. S." writes: May I say a word for "Grumbling Probationers"? I have much to do with their training, and I do not find they grumble more than any other class of women—or men, for that matter. Nurses had not, until lately, any paper in which to air their grievances. Now, thanks to the HOSPITAL, they can fully speak their minds. I have not found the fact of a woman being "a lady" makes her one bit less inclined to grumble. Until some power of divination is bestowed upon us, we must, in choosing our pros., always run a certain risk of getting a "grumbler" amongst the flock. However, give her a trial; tell her plainly, if she can't be contented, for the sake of herself as well as others, she must go. Much as one admires the work "Agnes Jones" began, I have always considered her a very foolish woman to kill herself. A good woman is much wanted, and should make her life spin out as long as she can. She, amongst the rest of us, took up the work either because she liked it or was obliged to do it, so, as Mr. James Adams once said, "There's not much credit either way." Perhaps the Plymouth pro. was not well, so extra work told on her, and I, with "One on the Active List," feel that any patient deserves great pity who has a jaded, weary nurse to look after him. Good nurses deserve all consideration, but if they grumble now and again try and find out the cause and remove it, but do not kill the goose with the golden eggs; in other words, do not expect too much. And one does get so tired! and though from my own experience very few nurses would refuse to give a few extra hours, still, many a good nurse would feel hardly used if asked to sit up all night after a hard day in the wards. It has to be done in private work, we know, and how many nurses work, and have worked, with little or no consideration; and of "gamps" I have known in former years I think "self-sacrifice" was not a prominent virtue, and really, after many years of hard work, I find "self-sacrifice" much talked of but little appreciated. Men and women in all grades grumble, and pros. are no worse than others.

SANITAS.

CHARGE NURSE E. WALSH writes: Will you kindly permit me to thank the Bethnal Green Sanitas Co. (Limited) for a parcel which I received from them to-day, containing samples of their different soaps and a canister of toilet powder? The excellence of these preparations makes them very useful on the Hospital Ships where I am now working.

### Princess Christian's Daughter.

MISS E. DURHAM, Farringford, Freshwater, Isle of Wight, acknowledges the following additional subscriptions towards a wedding present for Princess Louise of Schleswig-Holstein to be given as a proof of the gratitude of nurses for the interest Princess Christian has ever taken in their progress.

Matron A. R. M. Sharp, 5s.; C. McKay, 2s. 6d.; Superintendent M. G. C., 2s. 6d.; H. R. Ellinson, 2s. 6d.; Sister Armstrong, 2s. 6d.; Jane Glass, 2s.; Nurse Woodward, 2s.; J. Lindsay, 2s.; Nurse Horton, 2s.; Sister Edith, 1s.; T. and E. Connell, 2s.; Nurses (1s. each): M. Hicks, Fanny Davis, S. E. L., May White, Nurse Den, Margaret Peddie, H. L. White, Lavinia Sidy, M. E. Flatman, S. Carter, M. Neill, M. Blagney, E. Bellamy, A. C. Hobness (B.N.A.), Lucy J. Steel, L. Christian Watts, Fanny Arnold, Flora Macdonald, C. M. Martin, M. A. Wylie (B.N.A.), F. Barker, M. M. Buxton, Davis, Attree, V. H., Fillingham, Russel, Cassaide, Mary Hawthorn, M. Young (B.N.A.), Aryton, A Member of B.N.A..

On March 28th read Emma M. Dawe instead of Daws.

Total amount acknowledged in these pages, £20 15s. The names of the Committee and other particulars will be given later on.

### Appointments.

WOLVERHAMPTON INSTITUTION.—Miss Emma Loveys, who trained at Guy's, and has since taken charge of the Guy's Nurses' Home of Rest, Grove Park, has been appointed Lady Superintendent of the Queen Victoria Nursing Institution at Wolverhampton.

CROYDON INFIRMARY.—The following have been appointed charge nurses: Miss Annie Fielding, Mrs. Graves, Miss Margaret Henderson, and Miss Christina Kerr.

### Notes and Queries.

### Queries.

(3) *Stains.*—Is there any method of removing stains from water beds? —*Inquirer.*

(4) *Home Wanted.*—For a poor idiot boy, aged five years; must be Roman Catholic.—*Nurse Lucas.*

### Answers.

*Nora.*—You will find full particulars of all the institutions in Paris which employ English nurses, in THE HOSPITAL for November 1st, 1890.

*Barts.*—We have been obliged to defer the "Nursing Medals and Certificates" till the inquiry into the Eastern Hospital is concluded, but the medal of your school has been sketched, and will appear in due course.

*Mrs. S. K.*—Photos of the screen can be had from Messrs. Elliott and Fry, 55, Baker Street, Portman Square, W. We do not yet know at what price, but will tell you later.

*Sister Alice.*—We have no room for the lines sent.

*Photograph from Manchester.*—A cabinet photograph of a nurse has been received for the screen without name attached. Posted in Manchester on April 1st. Will the orginal please send her name?

*M. D.*—Miss Annie Chamberlain's address is 13, Reporton Road, Fulham. Her charges are the same as those of other masseuses.

*Nurse Lucas.*—The woman can be got into the Royal Albert Asylum, Lancaster, by votes of county subscribers.

*A Reader.*—We shall not publish your letter because you have not sent name and address. Besides we do not approve your sentiments, let us have the truth on small points, on all points, and no quibbles.

CORRECTIONS.—The Secretary of the City of London Lying-in Hospital writes, in reference to our article last week on "The Register of Nurses," to say that their certificate is given at the end of six weeks or two months' training, and not at the end of three weeks, as we stated. Our information was taken from the "Englishwoman's Year-Book," but we gladly make the correction.—Miss Shirley writes to say there are 102 nurses on the staff of the Staffordshire institution, whereas, on March 28th, it was stated in our pages, merely by a misprint, that the number was 69.

### Off Duty

### Catherine Fidelis.

"O, to be in England, now that April's there!"
Browning.

Drawn up under a shady tree close to, but not in, the Invalids' Walk was a bath-chair, in which lay back an indolent little figure. Passers-by, one and all, looked, some lingeringly, at the bright, sparkling face belonging to the invalid, and then their gaze glanced off to the spare, stern old military man on the seat, beside which the chair had halted. But neither little Cathie nor the General heeded the crowd; their world was a small one, containing but two inhabitants—themselves. Sometimes they spoke; then would follow the long, dreamy silences that are possible only between ourselves and those dearest to us.

"But you *are* better, Cathie? You're twice the little woman you were at that place, in the Engadine, where we spent the winter," and as he spoke the old soldier's steel-blue eyes were fixed, with what seemed an ever-present anxiety, upon the sweet face so dear to him.

"I love England, you see," was the quietly evasive answer, but Cathie laughed cheerily as she lovingly pulled the ears of Pat, the handsome collie that sidled up as close as he could to the bath-chair. The child was irrepressibly merry; the dreary disease that held her in its inexorable clutches could not master her light-heartedness; it was over her frail body alone that it held its direful dominion. Cathie had known for a considerable time that her earthly span was to be a short one; but death had no terrors for the little one, and she talked of it as a necessary journey. The young parents who had perished long since of cholera in India, leaving their baby to the General's care, were both waiting in that other country for her; it would be all right; Gran, of course, would be lonely just at first, but there was Pat to comfort him; and then, by-and-bye, he, too, would set out on the same journey. Oh, yes, it would be all right.

"And," continued the little speaker, "Bournemouth is the prettiest bit of England: we both think so, don't we?"

"Well, we'll stop here altogether," said the General, decisively.

"Altogether, oh yes! Then, presently, I'll go to sleep in the corner; I showed you the spot, Gran, that Sunday afternoon; and you and Pat must never go away; you'll stop here until its time for you to pack and come, too."

Cathie's listener writhed uneasily. The old soldier had seen plenty of active service. In the old days he, with the mad daring of an English boy, had led a forlorn hope up certain Indian steeps, a deed so doughty that the world rang with it—for a brief moment. But the veteran quailed before the thought of Cathie's journey. The little one saw the shrinking, and she went on merrily. "And, in case you should be idling, Gran, I'm going to leave you some work. You remember telling me, on my birthday, that you had given me all Mama's money?"

Yes, undoubtedly, the General remembered. Who had a better right than Cathie to the little fortune belonging to his pretty dead daughter and her mother?

"Well, that money is going to build a large cottage in one of the villages near here, where you said land was cheap; a cottage to hold six little ailing Catherines from anywhere. Nursie will look after them, it will keep her from fretting for me. Only they must be my namesakes; and you will call the cottage 'The Cat and Fiddle.' Don't you remember you told me that meant *Catherine Fidelis*, Gran?" and a ripple of happy laughter brought smile echoes to many a sombre face as the crowd passed and repassed through the enchanted dreamland, all a-glow with the freshness of April's dainty touches, which Bournemouth calls its pleasure-gardens.

In a village far away from the beautiful cliffs and chines of Bournemouth is an overgrown cottage, with the strange name of "The Cat and Fiddle" painted on its wooden gate—a quaint conceit, the meaning of which is known to few. The inmates are six little maids, always six, though not always the same.

There is a cheery atmosphere of "Better, to-day, thank you!" about the modest home, for the six ailing Catherines seldom fail to improve in health as rapidly as possible, to make room for a batch of successors, and Nursie's hands are too full to allow much grieving for the past. Two visitors never fail to come at regular periods, the General and Pat. The former finds the tiny establishment provides him with as much occupation in the shape of business as he can well manage in addition to his daily tramp on the breezy pier, his loiter through the Invalids' Walk, and his visits to the quiet corner where bright, happy little Cathie sleeps peacefully.

### To a Nurse.

Toil on! thou worker in a noble cause,
Nor heed the surging tide of life around;
Thou hast not time in thy short life to pause
When its horizon is by labour bound.

Toil on! and do not deem thy work in vain,
Though precious lives slip daily from thy grasp,
It may be those whom thou hast soothed in pain
Will, in a future world, thy kind hand clasp.

Toil on! unmindful of thy aching feet,
That have to-day paced wearily along.
In Paradise earth's labourer's soon shall meet
To swell the triumph of the victor's song.

Toil on! nor cease until thy nerveless hand
Drop to thy side—and thy life's work is o'er.
Thou shalt be called to the bright Angel-land
To rest with those above who toil no more!

M. G.

### Amusements and Relaxation.

**SPECIAL NOTICE TO CORRESPONDENTS.**

**Second Quarterly Word Competition commenced April 4th, ends June 27th, 1891.**

Competitors can enter for *all* quarterly competitions, but no competitor can take more than *one first prize* or *two* prizes of any kind during the year.

N.B.—Word dissections must be sent in WEEKLY not later than the first post on Thursday to the *Prize Editor*, 140, Strand, W.C., arranged alphabetically, with correct total affixed.

The words for dissection for this, the THIRD week of the quarter, being "MANIPUR."

| Names. | April 9th. | Totals. | Names | April 9th. | Totals |
|---|---|---|---|---|---|
| Christie | 21 | 21 | M. G. | 17 | 17 |
| Patience | 21 | 21 | Ivanhoe | 16 | 16 |
| Agamemnon | 21 | 21 | Weta | 16 | 16 |
| Hope | 20 | 20 | Lady Betty | 16 | 16 |
| Reldas | 20 | 20 | Mortal | 15 | 15 |
| Lightowlers | 2[illegible] | 20 | Little Eliza | 15 | 15 |
| Nurse J. S. | 19 | 19 | Dora | 15 | 15 |
| Qu'appelle | 19 | 19 | Ladybird | 14 | 14 |
| Jenny Wren | 18 | 18 | Psyche | 13 | 13 |
| Wyameris | 18 | 18 | Ugug | 13 | 13 |
| Paignton | 17 | 17 | Harrie | 10 | 10 |
| Theta | 17 | 17 | Grannie | 9 | 9 |
| Success | 17 | 17 | E[illegible]le | 9 | 9 |
| Tired | 17 | 17 | Grimalkin | 8 | 8 |

For Rules see The Hospital April 4th, 1891.

### Notice to Correspondents.

N.B.—Each paper must be signed by the author with his or her real name and address. A *nom de plume* may be added if the writer does not desire to be referred to by us by his real name. In the case of all prize-winners, however, the real name and address will be published.

## Figures and Tables

**Figure f1:**